# A robust enzymatic reporter system for the extremely thermophilic anaerobic bacterium *Anaerocellum bescii*

**DOI:** 10.3389/fmicb.2026.1652597

**Published:** 2026-01-29

**Authors:** Joey L. Galindo, Hansen Tjo, Jonathan M. Conway

**Affiliations:** 1 Department of Chemical and Biological Engineering, Princeton University, Princeton, NJ, United States; 2 Department of Molecular Biology, Princeton University, Princeton, NJ, United States; 3 Omenn-Darling Bioengineering Institute, Princeton University, Princeton, NJ, United States; 4 Andlinger Center for Energy and the Environment, Princeton University, Princeton, NJ, United States; 5 High Meadows Environmental Institute, Princeton University, Princeton, NJ, United States

**Keywords:** anaerobic thermophiles, *Anaerocellum bescii*, *Caldicellulosiruptor bescii*, enzymatic reporter, galactosidases, lignocellulose degradation, metabolic engineering, promoters

## Abstract

Thermophilic anaerobic organisms, particularly species that can naturally degrade lignocellulosic biomass, show great promise for next generation bioprocessing. This has led to the development of nascent genetic systems to metabolically engineer these non-model organisms. However, a major challenge remains a lack of reliable reporter systems compatible with the combination of thermophilic and anaerobic growth conditions. Additionally, native glycoside hydrolases in these organisms limit the usefulness of traditional glycosidic enzyme reporters (e.g., LacZ) because of the native background activity present on para-nitrophenyl glycoside substrates. Here we describe the development of a robust enzymatic reporter system that overcomes these challenges in *Anaerocellum* (f. *Caldicellulosiruptor*) *bescii*, an anaerobic, extremely thermophilic (T_opt_ ~ 78 °C), lignocellulolytic bacterium. Our method is based on heterologous expression of hyperthermophilic archaeal galactosidases: an ⍺-galactosidase from *Pyroccous furiosus* (*Pf*⍺gal), and a β-galactosidase from *Caldivirga maquilingensis* (*Cm*βgal). We show that these reporters produce strong, orthogonal signals on colorimetric substrates at high temperatures (≥90 °C) that eliminate background activity from endogenous galactosidases. We then demonstrate the capability of *Cm*βgal, the stronger of the two reporters, to distinguish differences in levels of expression between *A. bescii* promoter sequences, which we verify through qRT-PCR. With its high signal to noise ratio and relative ease of use, this reporter system offers a straightforward and robust method for assessing protein expression in *A. bescii* and potentially other anaerobic thermophilic organisms, opening doors to improved genetic tools and metabolic engineering applications for industrial biotechnology.

## Introduction

Reducing the world’s dependence on non-renewable and geographically limited fossil fuel-based feedstocks is a critical challenge. One promising alternative feedstock is plant biomass, especially its most common form, lignocellulose, which could provide an inexpensive and plentiful source of renewable energy and industrial chemicals (Lynd et al., 2022; Langholtz et al., 2024). The recalcitrance of lignocellulosic biomass severely limits its utilization through conventional bioprocessing approaches (Bing et al., 2021; Lynd et al., 2022). However, several thermophilic anaerobic bacteria are capable of natively breaking down lignocellulose, making them prime candidates for metabolic engineering (Blumer-Schuette et al., 2014; Lee et al., 2020; Bing et al., 2021). Yet, the genetic toolkits available in these non-model bacteria are still extremely limited, which has hampered engineering efforts (Loder et al., 2017; Blumer-Schuette, 2020). A major roadblock impeding the development of genetic tools in these thermophilic anaerobic bacteria is the lack of easily observable and background-free reporter systems that are compatible with the high temperature and oxygen-free growth conditions of these organisms (Loder et al., 2017; Riley and Guss, 2021; Streett et al., 2021).

*Anaerocellum* (f. *Caldicellulosiruptor*) *bescii* is the most thermophilic lignocellulose-degrading bacteria known, with an optimal growth temperature of 75–78 °C under anaerobic conditions (Lee et al., 2020). Development of genetic tools in this organism have enabled the metabolic engineering of *A. bescii*. These tools include deletions in the *pyr* locus (either Δ*pyrF* or Δ*pyrE*) to create uracil auxotroph strains that allows for positive selection with *pyr* gene complementation and counter selection on 5-FOA for marker replacement in *A. bescii* (Cha et al., 2013; Chung et al., 2013; Lipscomb et al., 2016). Positive selection is also available using a highly thermostable kanamycin resistance gene (*htk*) and selection on kanamycin antibiotic (Lipscomb et al., 2016). Using these tools, *A. bescii* has been successfully engineered to produce several industrially relevant products including ethanol, acetone, and 2,3-butanediol (Bing et al., 2024; Straub et al., 2020; Tanwee et al., 2023). However, the lack of robust, well-characterized genetic parts (e.g., promoters, reporters, terminators) as part of this genetic toolkit in *A. bescii* remains a major limitation to expanding metabolic engineering in it and similar organisms.

Control over protein expression is often most effectively achieved by varying the specific promoter and ribosome binding site (RBS) sequences upstream of a gene to change the level transcribed by RNA polymerase and translated by the ribosome, respectively (Kim et al., 2020; Riley and Guss, 2021). Yet, to date, expression of heterologous proteins in *A. bescii* has relied almost exclusively upon three native constitutive promoter-RBS sequences taken from directly upstream of the genes for the S-layer protein (P_slp_), a S30 ribosomal protein (P_S30_), and a bifurcating-hydrogenase (P_bh_) (Lee et al., 2020; Tanwee et al., 2023; Bing et al., 2024). All of these promoters are thought to drive relatively high expression, but there have been no direct comparisons of their strengths at the protein level (Lipscomb et al., 2016; Williams-Rhaesa et al., 2018; Lee et al., 2020). Furthermore, other methods of modulating transcription like CRISPRi, which has been demonstrated in other thermophiles, have yet to be implemented in *A. bescii* (Ganguly et al., 2020; Riley and Guss, 2021). A suitable anaerobic, extremely thermophilic protein reporter system would greatly enhance efforts to develop these and other genetic engineering tools in *A. bescii*.

Finding protein-based reporters that work well in anaerobic thermophiles has proven challenging because many reporter proteins permanently denature at the high native growth temperatures of thermophilic bacteria (Jensen et al., 2017; Kim et al., 2020; Riley and Guss, 2021; Hocq et al., 2023). Furthermore, many fluorescent or luminescent reporter proteins, such as GFP and luciferase, require oxygen to activate, and thus cannot be used in strict anaerobic conditions (Kim et al., 2020; Riley and Guss, 2021; Streett et al., 2021; Hocq et al., 2023). Though recently, by incubating previously grown cultures overnight under aerobic conditions, Ashok et al. (2025) utilized sfGFP as a reporter for promoter characterization in the anaerobic moderate thermophile *Acetivibrio thermocellus* (fm. *Clostridium thermocellum*), which like *A. bescii* is also highly efficient at degrading lignocellulose. Other fluorescent proteins like flavin mononucleotide (FMN)-binding fluorescent proteins (FbFPs), can fluoresce anaerobically under blue light but are quite dim compared to conventional fluorescent reporters (Kim et al., 2020; Riley and Guss, 2021; Streett et al., 2021). Another option is a class of protein tags which produce light upon binding to a small molecule ligand, the most notable of which are Snap-Tag, Clip-Tag, Halo-Tag, and Fluorescence-Activating Absorption-Shifting Tag (FAST) (Kim et al., 2020; Riley and Guss, 2021; Streett et al., 2021; Hocq et al., 2023). Yet, most of these tags are not thermostable enough to be used in extreme thermophiles like *A. bescii* (Mattossovich et al., 2020; Merlo et al., 2022; Hocq et al., 2023; Shin et al., 2025). The most promising *in vivo* demonstration of these fluorescent protein tags in an anaerobic thermophile was by Hocq et al. (2023) who expressed thermostable FAST-tag variants in the bacterium *Thermoanaerobacter kivui*; However, the reporter only functioned effectively up to 55 °C.

An alternative to fluorescent proteins is enzymatic reporters, such as the widely used *E. coli* β-galactosidase (*lacZ*) and β-glucuronidase (*gusA*) based systems, which detect protein expression indirectly by breaking down precursor molecules to a product with an colorimetric or otherwise easily quantifiable change (Kim et al., 2020; Riley and Guss, 2021; Streett et al., 2021). These systems have been used extensively in mesophilic anaerobes since many colorimetric molecules, like various ortho- or para-nitrophenol (pNP) linked compounds, do not require oxygen to produce a change in color (Jensen et al., 2017; Streett et al., 2021). A number of thermostable versions of these enzymes have been identified, but their implementation as reporters has remained limited (Honarbakhsh et al., 2012; Fujita et al., 2015; Jensen et al., 2017; Loder et al., 2017). This is in part because many thermophilic bacteria, particularly species that possess large inventories of lignocellulolytic enzymes, often express native versions of these enzymatic reporters or enzymes with identical activity, resulting in background activity that obscures any signal from the reporter (Honarbakhsh et al., 2012; Fujita et al., 2015). Thus, most attempts to implement enzymatic reporter systems in thermophiles have required time consuming deletions of the native enzyme from the genome or heterologous expression in species that do not produce background activity (Honarbakhsh et al., 2012; Fujita et al., 2015; Jensen et al., 2017; Loder et al., 2017; Li and Xu, 2025). Some notable attempts to implement enzymatic reporters in extreme thermophiles via these methods include expression of a β-glucuronidase in the archaeon *Sulfolobus solfataricus*, a β-glucosidase in the archaeon *Thermococcus kodakarensis*, a β-galactosidase in the bacterium *Thermus thermophilus*, and a β-galactosidase from *Geobacillus stearothermophilus* in *Geobacillus thermoglucosidasius* (Honarbakhsh et al., 2012; Fujita et al., 2015; Jensen et al., 2017; Li and Xu, 2025). In contrast, more straightforward attempts to implement an enzymatic reporter system have been achieved in the more moderately thermophilic *A. thermocellus* (T_opt_ ~ 55–60 °C), first by Olson et al. (2015) who used the aforementioned β-galactosidase from *G. stearothermophilus* for promoter characterization, and Liu et al. (2026) who primarily used the β-glucuronidase from *S. solfataricus* to develop an arabinose inducible promoter.

Here, we demonstrate a new reporter system in *A. bescii* using hyperthermophilic galactosidases: an ⍺-galactosidase from *Pyroccous furiosus* (*Pf*⍺gal, T_opt_ = 115 °C), and a β-galactosidase from *Caldivirga maquilingensis* (*Cm*βgal, T_opt_ = 110 °C) (van Lieshout et al., 2003; Letsididi et al., 2017). The optimal temperatures of these reporter enzymes are far above the temperature where native *A. bescii* enzymes are stable, thus enabling the elimination of background activity with a ≥ 90 °C incubation. The resulting reporter assay, consisting of a heat inactivation step followed by incubation with pNP-galactopyranoside substrate, produces a strong colorimetric signal while eliminating background from native enzymes. We demonstrate the utility of this reporter system by using it to compare the protein expression driven by two previously utilized *A. bescii* promoters. We validate that these protein expression results align with the transcriptional levels driven by these promoters. Together, this system offers a powerful reporter tool for the analysis of genetic parts and genetic manipulations in *A. bescii.* Furthermore, these reporters could easily be adapted for use in other lignocellulolytic, anaerobic, extreme thermophiles of interest as microbial chassis for industrial biotechnology.

## Materials and methods

### Bacterial strains and growth conditions

Plasmids were cloned in chemically competent *Escherichia coli* 10-beta (New England Biolabs) or TOP10 (Thermo Scientific). *E. coli* cultures were maintained at 37 °C in enriched Luria-Bertani (LB) medium (24 g/L yeast extract, 10 g/L tryptone, 5 g/L NaCl) or LB agar medium (5 g/L yeast extract, 10 g/L tryptone, 5 g/L NaCl, 15 g/L agar) plates with 50 μg/mL apramycin (Thermo Scientific). Unless described as otherwise, *A. bescii* strains were cultured in 50 mL of CM516 medium in 125 mL serum bottles sealed with 20 mm butyl rubber stoppers at 70 °C without shaking. CM516 medium follows the recipe for CC516 described previously by Lipscomb et al. (2016) with the only modification being a change in carbon source from 5 g/L cellobiose to 5 g/L maltose substrate. The CM516 medium was supplemented with 50 μg/mL kanamycin (IBI Scientific) as appropriate, and is referred to as CM516K medium. Sealed serum bottles containing sterile medium were made anaerobic through vacuum and gas cycling, with the headspace being replaced with 80% (v/v) N_2_ and 20% (v/v) CO_2_ gas. As is standard, *A. bescii* cell density was measured as the optical density at 680 nm (OD680) using a cuvette in a Nanodrop One C spectrophotometer (Thermo Scientific) with 1× DSM 516 salt solution used as the blanking solution (Lipscomb et al., 2016; Rodionov et al., 2021; Tjo et al., 2025).

### Vector construction

**Figure 1:**
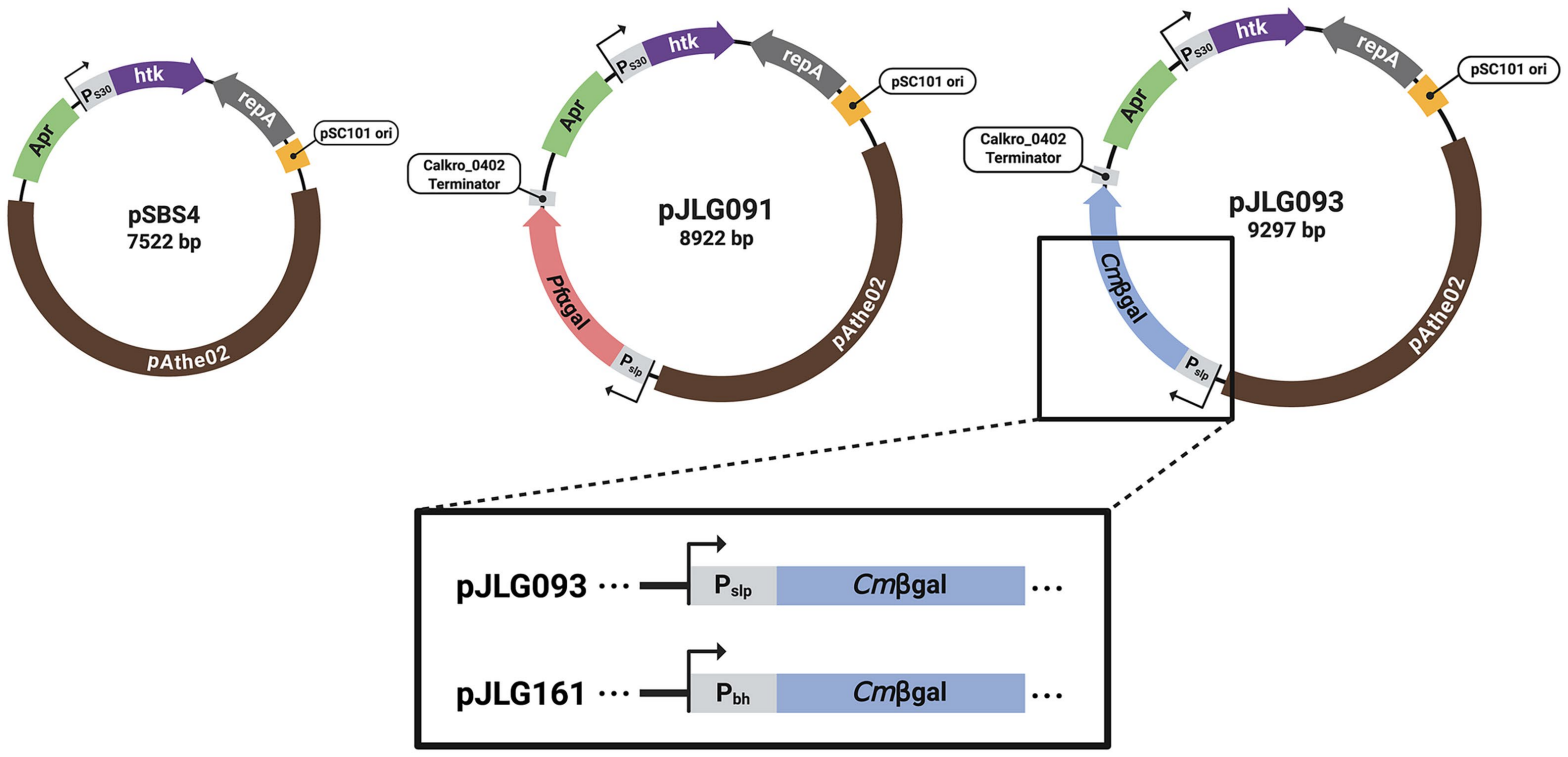
Maps of plasmids constructed and transformed into *A. bescii*. pSBS4 (empty vector) was used for the original implementation of the highly thermostable kanamycin (*htk*) selection marker in *A. bescii* (Lipscomb et al., 2016). This vector contains a native *A. bescii* replicating plasmid (pAthe02) which provides sequences necessary for replication in *A. bescii*, the *htk* gene expressed by P_S30_ for selection on kanamycin, as well as elements for cloning in *E. coli* including: an apramycin resistance marker (Apr), replication initiation protein A (repA), and the pSC101 origin. pSBS4 was modified to add a reporter expression site between Apr and pAthe02, resulting in: pJLG091 (P_slp_-*Pf*⍺gal), pJLG093 (P_slp_-*Cm*βgal), and pJLG161 (P_bh_-*Cm*βgal). Created in BioRender [Galindo (2025), https://BioRender.com/drjomi3].

Tables of oligonucleotide primers and synthesized DNA used to construct the plasmids in this study can be found in the Supplementary Tables S1,S2, respectively. The two promoter sequences used to express the reporter genes in this study consisted of the 200 bp sequences immediately upstream of the start codon of their associated gene (Table 1). These promoters were P_slp_ associated with the S-layer protein gene (*Athe_2303*), and P_bh_ associated with a bifurcating-hydrogenase gene (*Athe_1295*) (Table 1). Maps of plasmids constructed and utilized in this study are shown in Figure 1. pSBS4 (empty vector) was obtained from the lab of Dr. Robert Kelly (North Carolina State University) (Lipscomb et al., 2016). This vector consists of a native *A. bescii* replicating plasmid (pAthe02), the *htk* gene expressed by promoter P_S30_ associated the S30 ribosomal protein (Athe_2105), as well as elements that enable cloning in *E. coli* including: an apramycin resistance marker (Apr), replication initiation protein A (repA), and the pSC101 origin (Figure 1) (Chung et al., 2013; Lipscomb et al., 2016). Vectors pJLG091 and pJLG093 express the ⍺-galactosidase from *Pyroccous furiosus* (*Pf*⍺gal) and the β-galactosidase from *Caldivirga maquilingensis* (*Cm*βgal) respectively with P_slp_ (Figure 1). This expression site is based on the protein expression construct used previously in pJMC046 with the P_slp_ promoter and Calkro_0402 terminator, but is relocated on the pSBS4 backbone between Apr and pAthe02 (Conway et al., 2018). The backbone DNA for these vectors was PCR amplified from a sequenced plasmid that had been constructed previously via the insertion of a different P_slp_ driven gene into the pSBS4 backbone at this same site (Supplementary Table S1; Primers JLG021-22). Codon optimized genes flanked by appropriate overlapping regions were purchased (Twist Biosciences) for *Pf*⍺gal and *Cm*βgal (Supplementary Table S2) and assembled into plasmids via Gibson Assembly using the NEBuilder HiFi DNA Assembly kit (New England Biosciences). Vectors pSBS4 (empty vector), pJLG091 (P_slp_–*Pf*⍺gal), and pJLG093 (P_slp_–*Cm*βgal) were then cloned into chemically competent *E. coli* 10-beta, isolated using ZymoPURE miniprep kits (Zymo Research), and sequence confirmed (Azenta Genewiz). pJLG161 is identical to pJLG093 except that expression of *Cm*βgal is driven instead by P_bh_ (Figure 1). pJLG161 was constructed from pJLG093 in partnership with the Department of Energy Joint Genome Institute (JGI) at Lawrence Berkely National Lab (Berkely, CA) as described below. pJLG093 was first modified to create unique PmeI sites, aiding subsequent promoter insertion. The vector was linearized by PCR amplification (Supplementary Table S1; B431.093.VM.F & VM.R), and re-circularized via Gibson assembly together with an ultramer (Supplementary Table S1; JGI.UM1) purchased from Integrated DNA Technologies, using the NEBuilder HiFi DNA assembly kit. After validation of the modified vector, the sequence corresponding to P_bh_ was flanked by linkers designed for assembly into pJLG093_PmeI linearized by PmeI digest (Supplementary Table S2), purchased (Twist Biosciences) and assembled using the NEBuilder HiFi kit. These assemblies were subsequently transformed into chemically competent *E. coli* Top10 of which candidate colonies were picked, sequence verified on the Pacific Biosciences Revio platform (Pacific Biosciences), and analyzed using custom pipelines at the Joint Genome Institute. pJLG161 was subsequently isolated using ZymoPURE miniprep kits (Zymo Research), and sequence confirmed (Azenta Genewiz).

**Table 1:** Promoter sequences used to drive galactosidase reporter expression in *A. bescii.*

Promoter name	Associated gene	Sequence
S-layer protein promoter (P_slp_)	*Athe_2303*	acaggatttaaaagaggctatgcaggttttcaaagtgtaataaaattgtttcactaattttacagtttgattacagtttagtcagagctattgactattaaaaaaccgcttgataaaattttagctgtaagtgatgaggctataaaaaatagtataacctcatcactaaaaaatcatacaaggaggtttggtgagtagtt
Bifurcating hydrogenase promoter (P_bh_)	*Athe_1295*	tccattcctcagatgcccatcatctatgggagataaatgaaagggaattttttattgaaagtgatatactgtatacaatatttttcaattaaattctccaaaatttatacttcatttataacccgttgtatgctacaatattaacagtggttttaactccatatgttaaatttctaacaatagaagggggatgcagattt

### Plasmid preparation and transformation into *Anaerocellum bescii*

For transformation in *A. bescii*, larger quantities of plasmid DNA were extracted from *E. coli* using the ZymoPURE maxiprep kit (Zymo Research). Extracted plasmids were then methylated *in vitro* using the M.CbeI methyltransferase and purified via phenol-chloroform extraction as previously described (Chung et al., 2012; Lipscomb et al., 2016). Wild type *A. bescii* DSM 6725 was obtained from the lab of Dr. Robert Kelly (North Carolina State University). Competent *A. bescii* were grown on CM516 media containing amino acids (CM516-AA) to an optical density at 680 nm (OD680) of 0.04–0.08 and prepared for transformation as described previously (Lipscomb et al., 2016). Fifty microliters of competent cells were transformed with 1–2 μg of plasmid in a 1 mm gap electroporation cuvette using a Bio-Rad gene pulser at 1800 V, 400 Ω, and 25 μF. Electroporated cells were immediately resuspended in 1 mL of CM516 media and transferred to tubes containing 10 mL of the same media pre-heated to 70 °C. Cells were allowed to recover for 90 min before being transferred to pre-heated bottles containing 50 mL of selective CM516K media. After 24–36 h of growth, cells were passaged into 10 mL of fresh CM516K media and allowed to grow overnight. Passaged cells were then plated and grown for 48 h in solid selective CM516K media with 1.5% (w/v) agar at 70 °C under a 95% (v/v) N_2_ and 5% (v/v) H_2_ atmosphere in an anaerobic chamber. Single colonies were picked and screened via colony PCR (Supplementary Figure S1) using primers JLG181 and JLG224 (Supplementary Table S1). Presence of the correct promoter-reporter sequences were confirmed by long-read sequencing of colony PCR products (Azenta Genewiz, PCR-EZ).

### Enzymatic reporter assay

To detect galactosidase activity in *A. bescii* cells, para-nitrophenol-α-D-galactopyranoside (pNPαGal) and para-nitrophenol-β-D-galactopyranoside (pNPβGal) obtained from TCI chemicals, were used as colorimetric substrates. Substrate solutions contained 5 mM pNPαGal or pNPβGal dissolved in 100 mM sodium acetate pH 5.5 buffer which was chosen based on the previously determined optimal pH ranges of *Pf*⍺gal and *Cm*βgal (van Lieshout et al., 2003; Letsididi et al., 2017). *A. bescii* cells were prepared for galactosidase assays by first pelleting 5–15 mL of freshly grown cells at the maximum rotor speed (7,000 × g for 15 mL pellets or 21,000 × g for 5 mL pellets) for 10 min, followed by removal of the supernatant and storage at −80 °C for later use. Immediately prior to testing, cell pellets were resuspended and concentrated 3–5× in 1–3 mL of 100 mM pH 5.5 sodium acetate buffer to a final OD680 of 0.35–0.5, measured on a Nanodrop One C spectrophotometer with 100 mM sodium acetate buffer as the blanking solution. For assays involving heat-treatments, 50–100 μL of cells or blank buffer were aliquoted into PCR strip tubes and incubated in a thermocycler at 90 or 98 °C for 10 min unless described otherwise. To begin the reaction, 10–30 μL of cells or blank buffer were added to 60–80 μL of substrate solution or blank buffer to a total volume of 90 μL. Assays that involved wild type or the P_slp_–*Pf*⍺gal strain of *A. bescii* required 30 μL of cells, while testing of *Cm*βgal expressing *A. bescii* only required 10 μL of cells per reaction. Reactions were incubated in a thermocycler at the appropriate temperature for the experimentally prescribed time after which all reactions were immediately quenched with the addition of 180 μL of 1 M sodium carbonate. The absorbance at 405 nm (A405) of 100 μL of each reaction was then measured in a flat-bottomed clear 96 well plate using a BioTek SynergyH1 microplate reader (Agilent). For all reaction conditions the following controls were included: a substrate only (no cell) condition to account for the thermal background degradation of substrate, a no substrate condition for each cell type to account for background scattering from cellular debris, and a buffer only condition to isolate the absorbance due to debris in the prior control from the buffer itself. All reaction conditions were performed in technical triplicate.

Normalized galactosidase activity was calculated as defined in Equation 1 based on the equations in “Experiments in Molecular Genetics” for measuring β-galactosidase activity in *E. coli* using o-nitrophenyl-β-D-galactopyranoside (Miller, 1972). The most notable modifications to the quantification formula used by Miller (1972) are cellular debris background is explicitly accounted for with a series of control reactions rather than estimated with the absorbance at 550 nm, and normalization is done with the optical density at 680 nm (OD680) rather than that at 600 nm (OD600). In Equation 1, the A405 of the no cell control (A405_NC_) is subtracted from the A405 of the experimental condition (A405_exp_) to remove thermal background degradation of substrate. Separately, the A405 of the buffer only control (A405_BO_) is subtracted from that of the no substrate control (A405_NS_). This is then subtracted from the A405_exp_ − A405_NC_ difference to account for debris scattering. This final value is then divided by the previously measured OD680 of the resuspended *A. bescii* input to the assay to normalize for differences in the amount of cells added.


$$\text{Normalized Activity}=\frac{\left(A405_{\exp}-A405_{\mathrm{NC}})-(A405_{\mathrm{NS}}-A405_{\mathrm{BO}}\right)}{\mathrm{OD}680}$$ (1)

### Assessment of *Pf*⍺gal and *Cm*βgal as reporters in *Anaerocellum bescii*

To test for background activity from endogenous galactosidases, wild type *A. bescii* DSM 6725 were grown on CM516 media to an OD680 of 0.15 (late exponential), pelleted, and frozen. Cells were resuspended and heat-treated at 90 or 98 °C for 0, 10, 30, or 60 min prior to adding pNPαGal or pNPβGal solutions in the enzyme assay described above, with incubation for 2 h at 75 °C.

Prior to initial testing of the hyperthermophilic galactosidase reporters, *A. bescii* containing the empty vector (pSBS4), P_slp_–*Pf*⍺gal (pJLG091), and P_slp_–*Cm*βgal (pJLG093), were grown on selective CM516K media to an OD680 of 0.07–0.12 (mid-late exponential) and harvested as described above. To test the effects of various heat-treatment conditions on the reporters, resuspended empty vector, P_slp_–*Pf*⍺gal, and P_slp_–*Cm*βgal cells were heat-treated at 90 or 98 °C for 10 min which were subsequently added alongside un-heat-treated cells to both the pNP substrate solutions. For assays testing P_slp_–*Pf*⍺gal against the empty vector, cells were incubated for 2 h, while for assays testing P_slp_–*Cm*βgal, cells were incubated for 20 min. Signal to noise ratio was calculated as the normalized activity of reporter expressing cells on their respective preferred pNP substrates divided by that of the empty vector control at the corresponding conditions (i.e., P_slp_–*Pf*⍺gal/empty vector activity on pNP⍺Gal, or P_slp_–*Cm*βgal/empty vector activity on pNPβGal).

For time course experiments, cells that contained the empty vector or P_slp_–*Pf*⍺gal were prepared and heat-treated at 98 °C. Cells were then added to pNP⍺Gal solution and incubated for 0, 1, 2, or 3 h at 98 °C. Separately this was repeated for empty vector and P_slp_–*Cm*βgal cells except heat-treatments were carried out at 90 °C, cells were instead added to pNPβGal solution, and incubations were carried out at 90 °C for 0, 10, 20, or 30 min. For temperature optimization testing, cells that contained empty vector, P_slp_–*Pf*⍺gal, and P_slp_–*Cm*βgal were prepared and heat-treated at 90 °C which were subsequently added alongside un-heat-treated cells to their corresponding pNP substrate solutions (i.e., P_slp_–*Pf*⍺gal & empty vector on pNP⍺Gal; P_slp_–*Cm*βgal & empty vector on pNPβGal). These reactions were then incubated at the following temperatures: 75, 80, 85, 90, 95, and 98 °C. For this test, assays on pNP⍺Gal substrate were incubated for 2 h, while assays on pNPβGal were incubated for 10 min.

### Testing *Cm*βgal activity throughout the growth of *Anaerocellum bescii*

To start the growth curves of *A. bescii*, strains containing empty vector (pSBS4), P_slp_–*Cm*βgal (pJLG093), and P_bh_–*Cm*βgal (pJLG161) were inoculated at a target OD680 of 0.002 in 50 mL of selective CM516K media. Cultures were grown for 29 h in biological triplicate at 70 °C, with each culture’s OD680 measured at intervals of roughly 3–5 h. At 12, 18, 24, and 29 h, 4–5 mL of each culture was removed, after which cells were pelleted, frozen, and assayed as described above. To test for the activity of *Cm*βgal, thawed pellets were prepared as described above with heat treatment at 90 °C. Cells were then added to pNPβGal solution and incubated for 10 min at 90 °C.

### RNA extraction and qRT-PCR of *Cm*βgal expressing *Anaerocellum bescii*

Similar to previous studies, RNA was isolated from *A. bescii* containing pSBS4, pJLG093, and pJLG161 that were grown on CM516K media for 18 h to OD680 values of 0.11–0.16 (mid-late exponential phase), with three biological replicates for each strain (Williams-Rhaesa et al., 2018; Straub et al., 2020; Tanwee et al., 2023; Bing et al., 2024). After growth, 30–40 mL of cells were immediately pelleted at 6,000 × g for 10 min and, after removal of the supernatant, frozen at −80 °*C.* Prior to purification, thawed cell pellets were lysed as previously described with the addition of 240 μL of cold PBS, 75 μL of lysozyme (20 mg/mL), and 300 μL of the Monarch^®^ gDNA Tissue Lysis Buffer (New England Biosciences), followed by incubation at 37 °C for 15 min (Bing et al., 2024). Three hundred microliters of lystate from each pellet was then added to two volumes (600 μL) of Monarch^®^ StabiLyse DNA/RNA Buffer (New England Biosciences) (Bing et al., 2024). From this, RNA was purified using the Monarch^®^ Spin RNA Isolation Kit (New England Biosciences) as per the manufacturer’s instructions with the on-column DNase I treatment step. RNA concentrations were quantified using a Nanodrop One spectrophotometer (Thermo Scientific). qRT-PCR assays were carried out on a Viia7^™^ Real-Time PCR System (Thermo Scientific). qRT-PCR on extracted RNA samples was performed using the Luna^®^ Universal One-Step RT-qPCR Kit (New England Biosciences) according to the manufacturer protocol, with 50 ng of total RNA added to 10 μL reactions in a 384 well plate. A no-RT control condition was included for each experimental condition to check for DNA contamination. All reaction conditions, including for each biological replicate, were performed in technical triplicate. Expression of the *Cmβgal* gene (Supplementary Table S1; Primers JLG219-220) was calculated relative to that of the *A. bescii gapdh* (*Athe_1406*) using primers (Supplementary Table S1; Primers JLG211_CTS480-JLG212_CTS481) utilized in a previous *A. bescii* study (Straub et al., 2020).

## Results

### Implementation of two hyperthermophilic galactosidases as reporters in *Anaerocellum bescii*

**Figure 2:**
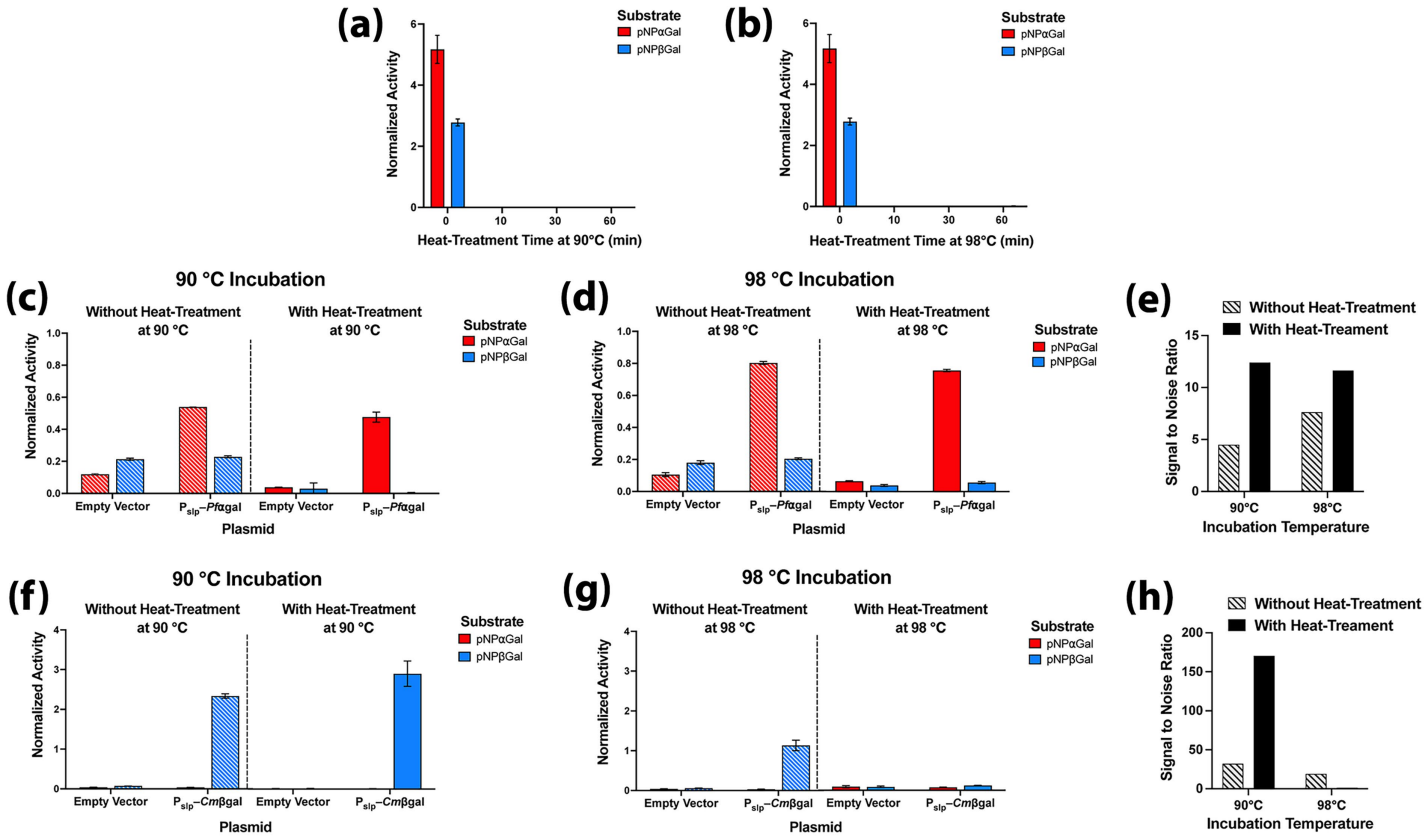
Initial development of reporter assay conditions. Native galactosidase activity of *A. bescii* is eliminated by heat-treatment. Activity from wild type *A. bescii* DSM 6725 cells on pNP⍺Gal (red) or pNPβGal (blue) as measured in a two-hour assay at 75 °C after being heat treated at: **(a)** 90 °C for 0, 10, 30 or 60 min; **(b)** 98 °C for 0, 10, 30 or 60 min. Assessing expression of *Pf*⍺gal and *Cm*βgal as hyperthermophilic galactosidase reporters in *A. bescii.* Here expression of both reporters is driven by P_slp_. Activity of *Pf*⍺gal vs. the empty vector strain on pNP⍺Gal and pNPβGal for: **(c)** 2 h at 90 °C with and without 10 min of heat-treatment at 90 °C; **(d)** 2 h at 98 °C with and without 10 min of heat-treatment at 98 °C. **(e)** The signal to noise ratio of *Pf*⍺gal on pNP⍺Gal, defined as the activity of *Pf*⍺gal divided by that of the empty vector strain after each of the four incubation conditions in **c,d**. Activity of *Cm*βgal vs. the empty vector strain on pNP⍺Gal and pNPβGal for: **(f)** 20 min at 90 °C with and without 10 min of heat-treatment at 90 °C; **(g)** 20 min at 98 °C with and without 10 min of heat-treatment at 98 °C. **(h)** The signal to noise ratio of *Cm*βgal on pNPβGal, defined as the activity of *Cm*βgal divided by that of the empty vector strain after each of the four incubation conditions in **f,g**. Error bars in all panels represent one standard deviation calculated from triplicate technical replicates at each reaction condition.

The genome of wild type *A. bescii* contains at least one characterized ⍺-galactosidase, along with several putative ⍺- and β-galactosidases (Lee et al., 2017; Drula et al., 2022). To assess the level of heat treatment needed to eliminate background activity from these enzymes on colorimetric pNP-glycoside substrates, prepared wild type *A. bescii* cells were heat treated at 90 or 98 °C for 0–60 min. Cells were then added to solutions of pNPαGal or pNPβGal and incubated for 2 h at 75 °C to test for endogenous ⍺- or β-galactosidase activity, respectively. Significant background activity was detected on both substrates with *A. bescii* cells that were not heat-treated (Figures 2a,b). However, this background activity was eliminated by heat treatment for as short as 10 min at either 90 or 98 °C (Figures 2a,b), indicating that native *A. bescii* ⍺- and β-galactosidases were inactivated with this relatively short incubation at temperatures above the organism’s optimal growth temperature.

To determine whether the hyperthermophilic galactosidases *Pf*⍺gal and *Cm*βgal could serve as effective enzymatic reporters under these conditions, wild type *A. bescii* DSM 6725 was transformed with plasmids pJLG091 (P_slp_–*Pf*⍺gal) and pJLG093 (P_slp_–*Cm*βgal), which drive strong constitutive expression of each galactosidase reporter with P_slp_ (Figure 1). A strain containing empty vector pSBS4 (Lipscomb et al., 2016) was also constructed as a control. *Pf*⍺gal and *Cm*βgal were selected because, among previously characterized hyperthermophilic galactosidases, they had the highest reported optimal temperatures, 115 °C and 110 °C, respectively, as determined by short *in vitro* assays with purified enzyme (van Lieshout et al., 2003; Letsididi et al., 2017). However, while *Pf*⍺gal is reported to be extremely thermostable (half-life of 15 h at 100 °C), *Cm*βgal is reported to lose all activity within 120 min at 95 °C and within 50 min at 100 °C (van Lieshout et al., 2003; Letsididi et al., 2017). Given the need to heat-treat *A. bescii* cells to eliminate native galactosidase activity and the differences between assay conditions, we evaluated the performance of both enzymes in our system.

Empty vector, P_slp_–*Pf*⍺gal, and P_slp_–*Cm*βgal cells were heat-treated at 90 or 98 °C for 10 min and tested alongside un-heat-treated cells at the same temperatures on both pNPαGal and pNPβGal (Figures 2c–h). In un-heat-treated samples, both reporters showed detectable activity above the empty vector control on their preferred substrates at 90 °C or 98 °C (left of Figures 2c,d,f,g). After a 10 min heat treatment at the assay temperature, background activity was reduced while preserving reporter activity in all cases except for *Cm*βgal at 98 °C, where the enzyme was fully inactivated during the 98 °C heat treatment (right of Figures 2c,d,f,g). For cases where the enzyme remains active, heat treatment improved signal to noise ratios (Figures 2e,h). Notably, *Cm*βgal at 90 °C showed a marked improvement in signal to noise ratio, increasing from 32 times to 170 times background with heat treatment (Figure 2h). Signal to noise for *Pf*⍺gal assays are also improved with heat treatment (Figure 2e), but much more modestly due to its weaker activity in general, though this enzyme does retain function under our assay conditions at both 90 and 98 °C (Figures 2c–e). Finally, the two reporters act orthogonally, with no detectable activity above that of the empty vector control detected on their non-preferred substrates (Figures 2c,d,f,g). Because heat treatment at 90 °C for 10 min achieved improvements in signal to noise ratio while maintaining activity of both enzymes, this heat treatment was chosen as the standard in subsequent assays.

**Figure 3:**
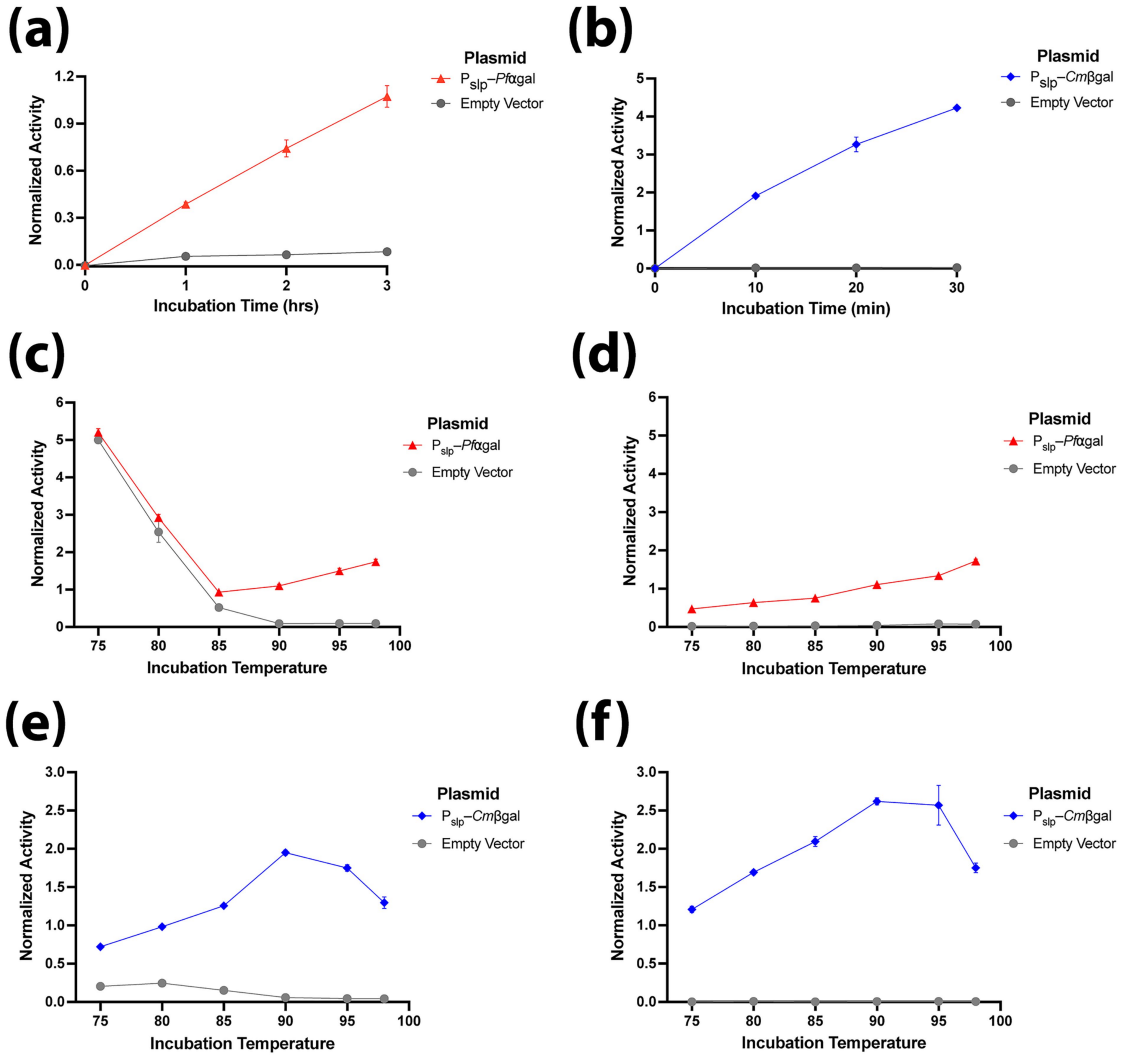
Optimization of incubation conditions for *Pf*⍺gal and *Cm*βgal. Here expression of both reporters is driven by P_slp_. Activity detected after various incubation times with pNP substrate compared with the empty vector strain for: **(a)** *Pf*⍺gal on pNP⍺Gal for 0–3 h at 98 °C with 10 min of heat-treatment at 98 °C; **(b)** *Cm*βgal on pNPβGal for 0–30 min at 90 °C with 10 min of heat-treatment at 90 °C. Activity detected after incubation at various temperatures of *Pf*⍺gal on pNP⍺Gal compared with the empty vector strain: **(c)** incubation for 2 h at 75–98 °C without heat-treatment; **(d)** incubation for 2 h at 75–98 °C with 10 min of heat-treatment at 90 °C. Activity detected after incubation at various temperatures of *Cm*βgal on pNPβGal compared with the empty vector strain: **(e)** incubation for 10 min at 75–98 °C without heat-treatment; **(f)** incubation for 10 min at 75–98 °C with 10 min of heat-treatment at 90 °C. Error bars in all panels represent one standard deviation calculated from triplicate technical replicates at each reaction condition.

Next, the optimal reporter assay conditions were evaluated. To ensure a sufficiently strong signal while maintaining approximately linear behavior with respect to incubation time, we measured enzymatic activity as a function of assay duration on heat-treated cells (Figures 3a,b). These assays showed that a 2–3 h incubation was appropriate for Pslp–*Pf*⍺gal on pNPαGal (Figure 3a), whereas a 10–20 min incubation was appropriate for Pslp–*Cm*βgal on pNPβGal (Figure 3b). To optimize assay temperature, assays were conducted on the reporter strains versus the empty vector control on their respective substrates from 75–98 °C with and without heat-treatment at 90 °C for 10 min (Figures 3c–f). *Pf*⍺gal produced the strongest signal at 98 °C (the highest reaction temperature we could reliably achieve in our thermocycler), and heat treatment was essential for eliminating the significant native ⍺-galactosidase activity at incubation temperatures below 85 °C (Figures 3c,d, without and with heat treatment, respectively). *Cm*βgal showed optimal activity at 90 °C, with decreasing activity at higher temperatures (Figures 3e,f). While background activity without heat treatment was much lower for pNPβGal (Figure 3e) than pNPαGal (Figure 3c), likely due to the shorter assay incubation time, heat treatment still eliminated nearly all native β-galactosidase activity (Figures 3e,f). While *Cm*βgal produced stronger signals than *Pf*⍺gal, its activity decreased at temperatures above 90 °C in the conditions of our assays (Figures 2f,g, 3e,f). This thermostability profile is in line with prior characterization of *Cm*βgal, which showed *in vitro* activity at its T_opt_ of 110 °C in a 2 min assay began to diminish immediately when the enzyme was pre-incubated at 100 °C, while no loss in activity was observed after incubation at 90 °C for 2 h (Letsididi et al., 2017). Considering its superior signal to noise ratio and stronger normalized activity in shorter incubation times, *Cmβ*gal was chosen as the reporter for subsequent tests of expression in *A. bescii*.

### Utilizing *Cm*βgal to distinguish differences in protein expression in *Anaerocellum bescii*

**Figure 4:**
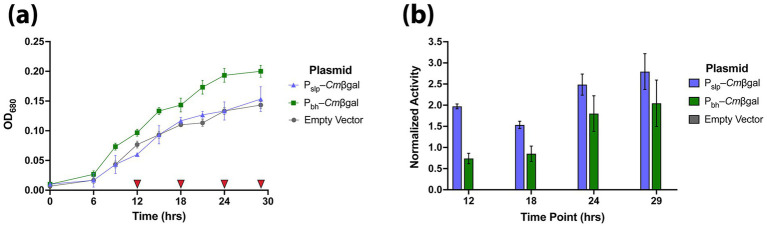
**(a)** Growth of *A. bescii* strains transformed with plasmids expressing *Cm*βgal with P_slp_ or P_bh_ as well as the empty vector strain over the course of 29 h. Red triangles (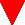) indicate time points (12, 18, 24, and 29 h) where cells were harvested for enzyme assays. **(b)** Corresponding normalized β-galactosidase activity of prepared *A. bescii* cells at 12, 18, 24, and 29 h. Cells were heat-treated for 10 min at 90 °C followed by another 10-min incubation at 90 °C on pNPβgal. Error bars in both **(a,b)** represent one standard deviation between triplicate biological replicates.

Next, reporter expression was tested over the growth of *A. bescii* strains containing the *Cm*βgal reporter under the control of two previously utilized promoters, P_slp_ and P_bh_ (Table 1). Based on previous studies, the P_bh_ promoter should drive somewhat lower expression than P_slp_ (Williams-Rhaesa et al., 2018). *A. bescii* strains containing pSBS4 (empty vector), pJLG093 (P_slp_–*Cm*βgal), and pJLG161 (P_bh_–*Cm*βgal) were grown and monitored over the course of 29 h in biological triplicate (Figure 4a). At time points of 12, 18, 24, and 29 h, corresponding roughly to exponential, late exponential, early stationary, and stationary growth phases, respectively, cells were harvested for enzyme reporter measurement on pNPβGal with heat-treatment and incubation at 90 °C (Figure 4b). As expected, no significant activity was detected from the empty vector strain at any stage of growth (Figure 4b). The relative activity of the two promoters vary over the course of cell growth phase with P_bh_–*Cm*βgal having 37, 56, 72, and 73% the activity of P_slp_–*Cm*βgal at 12, 18, 24, and 29 h of growth, respectively (Figure 4b). In general, activity from both *Cm*βgal expressing strains appears to increase as *A. bescii* enters stationary phase (24- and 29-h timepoints), though variability between biological replicates also increases in stationary phase (Figure 4b).

**Figure 5:**
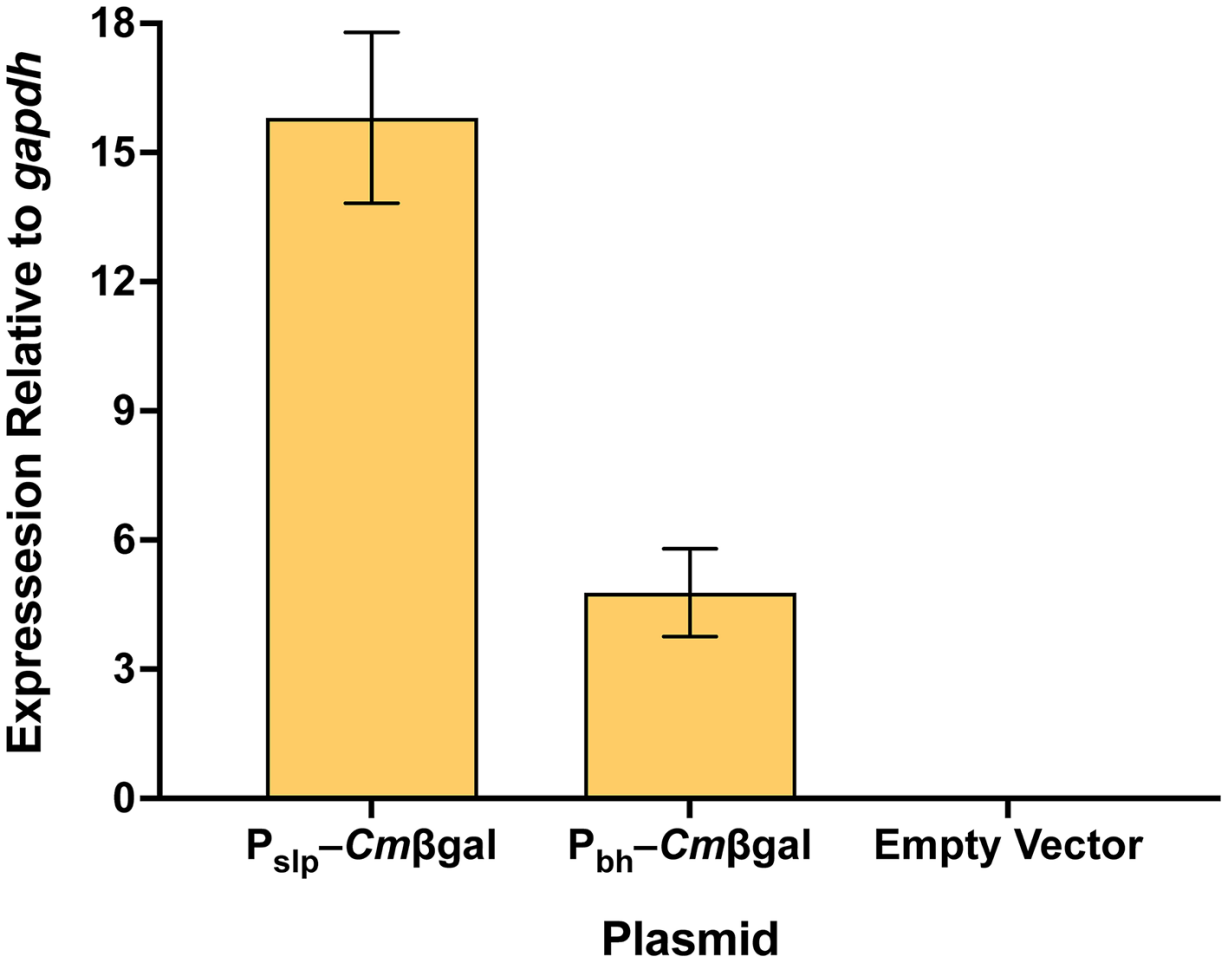
Transcription of the *Cm*β*gal* reporter gene in *A. bescii* strains grown for 18 h, relative to that of the endogenous *gapdh* (*Athe_1406*) housekeeping gene. Error bars represent one standard deviation across biological triplicates.

To assess how the *Cm*βgal reporter activity levels mirror transcript levels, qRT-PCR was performed on the *Cm*βgal gene. RNA was extracted from empty vector, P_slp_–*Cm*βgal, and P_bh_–*Cm*βgal *A. bescii* strains in late exponential phase (18-h timepoint) grown in biological triplicate. Levels of *Cm*βgal transcription in each strain were calculated relative to that of the endogenous *A. bescii* glyceraldehyde-3-phosphate dehydrogenase *gapdh* (*Athe_1406*) housekeeping gene as is standard in the literature (Williams-Rhaesa et al., 2018; Straub et al., 2020; Tanwee et al., 2023). Results show that both P_slp_ and P_bh_ drive strong levels of transcription, with expression of 15.8× and 4.8× that of *gapdh,* respectively (Figure 5). P_slp_ is the stronger promoter with an average level of transcription 3.3× that of P_bh_ (Figure 5). This mirrors a smaller difference in enzyme activity, where *Cm*βgal expressed by P_slp_ produced an average enzyme activity 1.8× that driven by P_bh_ (Figure 4b).

## Discussion

In this work we demonstrate the use of two hyperthermophilic galactosidases, *Pf*⍺gal and *Cm*βgal, as enzymatic reporters in *A. bescii.* These reporters and the activity assay we develop here expand the toolbox for assessing protein expression levels in *A. bescii*. In this assay, heat-treatment for as short as 10 min at 90 °C is sufficient to eliminate any activity from endogenous *A. bescii* galactosidases, reducing background and improving reporter signal to noise (Figure 2). The *Pf*⍺gal and *Cm*βgal reporters function orthogonally, with no activity above background on their non-preferred pNP substrate (Figures 2c,d,f,g), opening the possibility that dual expression within a single strain could be used to produce distinct readouts. This could prove useful for certain applications such as engineering transcriptional terminators. Of these two reporter enzymes, *Cm*βgal is the stronger reporter with a far greater dynamic range in shorter assay time. While *Pf*⍺gal requires incubations on the order of several hours at an optimal temperature 98 °C, *Cm*βgal produces ~10× the signal of *Pf*⍺gal relative to the empty vector with fewer cells in 10 min at an optimal incubation temperature of 90 °C (Figures 2c–h, 3).

By expressing *Cm*βgal with two different promoters, P_slp_ and P_bh_, we show that our hyperthermophilic β-galactosidase based reporter system can measure differential expression in *A. bescii* (Figures 4a,b). The strength of both promoters appears to increase but become more variable as cells enter stationary phase (Figure 4b). Because the *in vivo* protein turnover rate of *Cm*βgal in *A. bescii* is unknown, it is possible that this apparent increase in expression in stationary phase is due to accumulation of reporter protein as cell division slows. Additionally, reporter stability or turnover may be affected by changes in growth conditions or during cellular stress, so users should evaluate reporter behavior under their application-specific conditions. While P_slp_ consistently drives higher enzymatic expression as measured by reporter activity, P_bh_ does seem to increase in relative strength in stationary phase (Figure 4b). To qualitatively confirm the relative strengths of these promoters, we performed a qRT-PCR comparison. At the sampled late exponential timepoint (18 h), P_slp_ transcript levels were approximately 3.3× higher than P_bh_ (Figure 5), consistent with prior reports of a 3–6× difference (Williams-Rhaesa et al., 2018). Enzymatic activity differed by a smaller margin at this same timepoint with enzymatic activity from expression driven by P_slp_ being 1.8× that of P_bh_ (Figure 4b). Differences in these output levels at the transcript and activity levels likely reflect combined transcriptional, translational, and post-transcriptional processes. Thus, like other enzymatic reporters, this system would likely be most useful for end-point assays that detect historic rather than real-time levels of protein expression (Riley and Guss, 2021; Streett et al., 2021).

The promoter regions used ahead of the reporter genes in this study are the native 200 bp immediately upstream of their associated genes (Table 1). Following past work in *A. bescii*, they include the native ribosome binding sites (RBSs) associated with each gene. Consequently, the differences we observe between P_slp_ and P_bh_ reflect the combined influence of transcriptional and translational elements in these 200 bp, rather than promoter strength alone. Modulating the protein expression level of genes of interest in bacteria requires considering regulatory contributions across multiple levels of the central dogma including transcription and translation (Kent and Dixon, 2020). Although the reporter assay here does not deconvolute these individual contributions, it demonstrates that *Cm*βgal reliably distinguishes the overall enzymatic activity output driven by commonly used native regulatory sequences in *A. bescii*. In the future, this system could be applied to independently characterize genetic parts such as native or synthetic promoters, RBS elements, terminators, or combinations of these elements.

Additionally, cell growth was not detrimentally affected by expression of *Cm*βgal, with both reporter-expressing lines growing as well or better than the empty vector control (Figure 4a), indicating the reporter is non-toxic in *A. bescii*. Furthermore, given that *A. bescii* natively produces a wide variety of carbohydrate active enzymes (CAZymes), including several galactosidases, and the fact that galactose is relatively scarce in the typical lignocellulosic substrates consumed by *A. bescii* as well as the maltose-based media used in this study, we do not expect a significant impact on cellular metabolism due to expression of these reporters (Lee et al., 2017, 2020; Rodionov et al., 2021; Drula et al., 2022).

Taken as a whole, we describe an easy to perform and robust enzymatic reporter system in *A. bescii*. This system should be broadly useful for future genetic tool development, strain identification, and gene expression analysis. While demonstrated here in *A. bescii*, we expect this hyperthermophilic enzyme reporter system could easily be adapted for use in other thermophilic anaerobic species, and would be especially valuable in species that grow at temperatures >70 °C where other anaerobic reporters are not viable or that possess native glycosidase enzymes that obscure less thermophilic enzymatic reporters. Ultimately this reporter system will enable the development of new genetic tools, metabolic engineering approaches, and next generation bioprocessing efforts using anaerobic thermophiles.

## Data Availability

The original contributions presented in the study are included in the article/Supplementary material, further inquiries can be directed to the corresponding author.
