# Supplementary material for: A robust enzymatic reporter system for the extremely thermophilic anaerobic bacterium Anaerocellum bescii
Source: Front Microbiol. 2026 Jan 29;17:1652597. doi: 10.3389/fmicb.2026.1652597 (PMC12895679; doi:10.3389/fmicb.2026.1652597)
Supplement: Supplementary file 1 [file Data_Sheet_1.pdf]

Supplementary Information for:

A Robust Enzymatic Reporter System for the Extremely Thermophilic Anaerobic Bacterium *Anaerocellum bescii*

Joey L. Galindo [1], Hansen Tjo[1], Jonathan M. Conway [1,2,3,4,5,*]

[1] Department of Chemical and Biological Engineering, Princeton University, Princeton, NJ 08544, USA

[2] Department of Molecular Biology, Princeton University, Princeton, NJ 08544, USA

[3] Omenn-Darling Bioengineering Institute, Princeton University, Princeton, NJ 08544, USA

[4] Andlinger Center for Energy and the Environment, Princeton University, Princeton, NJ 08544, USA

[5] High Meadows Environmental Institute, Princeton University, Princeton, NJ 08544, USA

*** Correspondence:**
Jonathan M. Conway
jmconway@princeton.edu

Table S1. Oligonucleotide primers and ultramers used in this study.

**Primer Name**	**Sequence (5'→3')**	**Use**
JLG021	aactactcaccaaacctccttg	pJLG091 and pJLG093 construction
JLG022	taaaggaggactataataaagggagc	pJLG091 and pJLG093 construction
JLG181	gaatctcgagtcttctgacgct	Colony PCR of pSBS4 based vectors
JLG224	gtgagttatacacagggctgg	Colony PCR of pSBS4 based vectors
JLG211_CTS480	tcttgctctccttaaatccttgtg	qPCR-*A. Bescii gapdh* gene (Athe_1406)
JLG212_CTS481	ggtgtaaatgaggagatgtacgac	qPCR-*A. Bescii gapdh* gene (Athe_1406)
JLG219	gatatggatatggatgcgaaagaaac	qPCR-*Cm*βgal reporter gene
JLG220	ccaaataatatggtctctgataatctgc	qPCR-*Cm*βgal reporter gene
B431.093.VM.F	atggatatttcttttccaaaatct	pJLG093_PmeI and pJLG161 construction
B431.093.VM.R	agcgtcagaagactcgagat	pJLG093_PmeI and pJLG161 construction
JGI.UM1	gtcaaaaaacggtgcgcttactgttcgtgggctcatgggaatct cgagtcttctgacgctgtttaaacatggatatttcttttccaaaatc ttttagatttggatggtctcaggcaggatttcagtct	pJLG093_PmeI and pJLG161 construction

Table S2. Synthesized genes and promoters used in this study.

**Name/description**	**Sequence (overlap regions in bold underline)**
Codon optimized ***Pf*αgal** gene with overlaps for gibson assembly	**caaggaggtttggtgagtagtt**atgagagcattggtttttcatggaaacttgcagtatgcagaaattccaaaatctgaaattccaaaagttattgaaaaagcatattttccaacaatttctgaattgattagaagagaaattccatttggattgaacattacaggatattctttgtctttttgccaaaagatttgattgcattgattaaagaaggaattgaatctggattgattgaaattttgggaacatcttatacacatgcaattttgccattgttgccattgtctagagttgaagcacagattaaaagagatagagaagttaaagaaaacatttggaagtttctccagaaggattttggttgccagaattggcatatgatccaattattccagcaattttgagagataacaactatgaatatttgtttgcagatggagaagcaatgttgttttctaaccatttgaactctgcaattaaaccaattaaaccattgtatccacatttgattaaagcacagagaggagaaggattggtttatttgaactatttgttgggattgagagaattgaaaaaagcaattaacttggtttttgaaggaaaagttacattggaagcagttaaagaaattgaagcaattccagtttgggtttctattaacacagcagttatgttgggagcaggaagatttccattgatgaacccaaaaaaagttgcaaaatgggttaaagaaaaagatgaaattttgttgtatggaacagatattgaatttttgggatatagagatattgcaggatataaaattacaatttctaacttgttggaaattattaacgaattggaaggagaattgggattgccaagaaaaattaaacattctgaaaaaaaattgtatttgagaacatcttcttgggcaccagataaatctttgagaatttggacagaagatgaaggaaacgcaagattgaacatgttgacatcttatatggatggagaattggcatttttggcagaaaactctgatgcaagaggatgggaaccattgccagaaagaagattggatgcatttaaagcaatttatacacattggagatctgaaaacggaaaacatcatcaccaccaccaccat**taaaggaggactataataaagggagc**
Codon optimized ***Cm*βgal** gene with overlaps for gibson assembly	**caaggaggtttggtgagtagtt**atggatatttcttttccaaaatcttttagatttggatggtctcaggcaggatttcagtctgaaatgggaacaccaggatctgaagatccaaacacagattggtatgtttgggttcatgatccagaaaacattgcatctggattggtttctggagatttgccagaacatggaccaggatattggggattgtatagaatgtttcatgataacgcagttaaaatgggattggatattgcaagaattaacgttgaatggtctagaatttttccaaaaccaatgccagatccaccacagggaaacgttgaagttaaaggaaacgatgttttggcagttcatgttgatgaaaacgatttgaaaagattggatgaagcagcaaaccaggaagcagttagacattatagagaaatttttctgatttgaaagcaagaggaattcattttattttgaacttttatcattggccattgccattgtgggttcatgatccaattagagttagaaaaggagatttgtctggaccaacaggatggttggatgttaaaacagttattaactttgcaagatttgcagcatatacagcatggaaatttgatgatttggcagatgaatattctacaatgaacgaaccaaacgttgttcattctaacggatatatgtgggttaaatctggatttccaccatcttatttgaactttgaattgtctagaagagttatggttaacttgattcaggcacatgcaagagcatatgatgcagttaaagcaatttctaaaaaaccaattggaattatttatgcaaactcttcttttacaccattgacagataaagatgcaaaagcagttgaattggcagaatatgattctagatggatttttttgatgcaattattaaaggagaattgatgggagttacaagagatgatttgaaaggaagattggattggattggagttaactattattctagaacagttgttaaattgattggagaaaaatcttatgtttctattccaggatatggatatggatgcgaaagaaactctatttctccagatggaagaccatgctctgattttggatgggaattttatccagaaggattgtatgatgttattatgaaatattggtctagatatcatttgccaatttatgttacagaaaacggaattgcagatgcagcagattatcagagaccatattatttggtttctcatatttatcaggtttatagagcaattcaggaaggagcaaacgttaaaggatatttgcattggtctttgacagataactatgaatgggcatctggattttctatgagatttggattgttgcaggttgattattctacaaaaaaacagtattggagaccatctgcatatgtttatagagaaattgcaaaatctaaagcaattccagaagaattgatgcatttgaacacaattccaccaacaagatctttgagaagacatcaccaccaccaccat**taaaggaggactataataaagggagc**
Bifurcating hydrogenase promoter (**P_{bh}**) with overlaps for gibson assembly	**gctcatgggaatctcgagtcttctgacgct**tccattcctcagatgcccatcatctatgggagataaatgaaagggaattttttattgaaagtgatatactgtatacaatatttttcaattaaattctccaaaatttatacttcatttataacccgttgtatgctacaatattaacagtggttttaactccatatgttaaatttctaacaatagaaggggggatgcagattt**atggatatttcttttccaaaatctttaga**

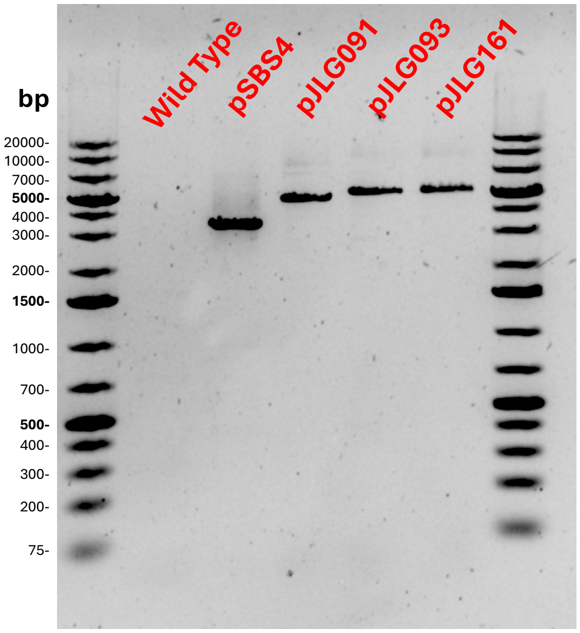

Figure S1. Colony PCR of strains used in this study (**Table S1; Primers JLG181 & 224**) . Wild type *A. bescii* DSM 6725 cells show no amplification as expected. Observed bands align with expected amplificon sizes of 3.3 kb for pSBS4, 4.7 kb for pJLG091, and 5.1 kb for pJLG093 and pJLG161.
